# QT interval prolongation and heart failure severity: A longitudinal study

**DOI:** 10.6026/973206300221175

**Published:** 2026-02-28

**Authors:** Mani Shankar Pandey, Jyoti Mani Shankar Pandey

**Affiliations:** 1 Department of Cardiology, Nalanda Medical College and Hospital, Patna, Bihar, India; 2 Department of Ophthalmology, Nalanda Medical college and Hospital, Patna, Bihar, India

**Keywords:** Arrhythmic events, heart failure, left ventricular ejection fraction, mortality, QT interval prolongation

## Abstract

Heart failure (HFrEF or HFpEF) patients are at risk for arrhythmic events and mortality, yet the role of QT interval prolongation as a prognostic marker in this population remains poorly understood. Patients with heart failure (HFrEF or HFpEF) were assessed for changes in QT interval, NYHA class, LVEF, BNP levels and hospitalizations. Results showed significant associations between prolonged QT interval and worsening heart failure, with increased risks of arrhythmic events and mortality. The advancement to knowledge in this study is the identification of QT interval prolongation as a significant prognostic marker for heart failure severity.

## Background:

Heart failure (HF) is a clinical syndrome characterized by the heart's inability to pump blood effectively, leading to inadequate tissue perfusion and oxygenation. It affects millions worldwide and its prevalence continues to rise due to factors such as aging populations and increasing rates of coronary artery disease, hypertension and diabetes [1]. HF is typically classified into two main categories: heart failure with reduced ejection fraction (HFrEF) and heart failure with preserved ejection fraction (HFpEF). Despite advances in treatment, HF remains a major cause of morbidity, mortality and hospitalization, making it a significant public health burden [2]. Electrocardiographic (ECG) abnormalities are common in HF patients and one of the most notable findings is the prolongation of the QT interval. The QT interval represents the time taken for the heart's electrical system to complete one cycle of depolarization and repolarization of the ventricles [3]. A prolonged QT interval is generally considered to be a sign of delayed repolarization, which can predispose to potentially life-threatening arrhythmias, including torsades de pointes and ventricular fibrillation. QT interval prolongation has been well-established as a predictor of sudden cardiac death in various populations, including those with heart failure [4]. The QT interval has been shown to be prolonged in patients with HF and this prolongation tends to correlate with worsening heart failure severity. However, the exact relationship between QT prolongation and the progression of heart failure remains unclear [5]. Previous studies have indicated that QT interval prolongation in HF may be influenced by a variety of factors, including autonomic dysfunction, electrolyte imbalances (e.g., hypokalemia, hypomagnesemia), the use of certain medications (e.g., diuretics, anti-arrhythmic drugs) and changes in the heart's structural and electrical properties [6]. QT prolongation can also be exacerbated by comorbidities, including ischemic heart disease, diabetes and hypertension, all of which are common in HF patients [7]. QT prolongation in HF has been associated with a higher risk of arrhythmias, hospitalization and death, which underscores the importance of closely monitoring the QT interval in these patients [8]. However, while it is known that prolonged QT intervals are linked to adverse outcomes, the degree of correlation between QT prolongation and the severity of HF remains a subject of ongoing debate [9]. Some studies have suggested that the severity of heart failure, as measured by parameters such as left ventricular ejection fraction (LVEF), New York Heart Association (NYHA) class and BNP levels, may influence the extent of QT prolongation. Other studies have shown that QT prolongation is more pronounced in patients with advanced HF, particularly those with HFrEF [10]. Despite these observations, there is still limited prospective data on the longitudinal relationship between QT interval prolongation and the progression of heart failure over time [11]. Understanding whether QT prolongation serves as an early marker of worsening heart failure or as a predictor of adverse outcomes in HF patients could significantly enhance clinical decision-making, particularly in the management of high-risk patients [12]. Therefore, it is of interest to determine the correlation of QT interval prolongation with the severity of heart failure over time. A longitudinal study examining this relationship could provide critical insights into the potential of QT interval prolongation as a prognostic marker for heart failure progression, helping guide treatment strategies and improve patient outcomes.

## Methodology:

This study was a prospective longitudinal cohort study at Department of Cardiology, Nalanda Medical College and Hospital, Patna, Bihar, India for 12 months designed to investigate the correlation between QT interval prolongation and the severity of heart failure over time. Patients diagnosed with heart failure were tracked and their QT intervals were measured at baseline and during regular follow-up intervals to assess changes over time. The primary aim was to explore whether QT prolongation correlated with worsening heart failure severity, as measured by clinical markers such as New York Heart Association (NYHA) class, left ventricular ejection fraction (LVEF), brain natriuretic peptide (BNP) levels and hospitalizations. The study included patients aged 18 years and older who had a diagnosis of heart failure, either with reduced ejection fraction (HFrEF) or preserved ejection fraction (HFpEF), according to current clinical guidelines. Exclusion criteria included patients with a history of congenital QT syndrome or other inherited arrhythmia syndromes, those with active infections or severe comorbidities (e.g., acute myocardial infarction), patients who had undergone a heart transplant and those unable or unwilling to provide informed consent. Both inpatients and outpatients diagnosed with heart failure were included to ensure a representative sample from various stages of the disease. Data were collected from each participant on demographic and clinical characteristics, including age, gender, medical history, comorbidities (e.g., hypertension, diabetes), medication use and lifestyle factors (e.g., smoking, alcohol consumption). Clinical measures of heart failure severity, such as NYHA functional class, LVEF (obtained through echocardiography) and BNP levels were also recorded. Electrocardiograms (ECGs) were obtained at baseline and at each follow-up visit to measure the QT interval, which was assessed using lead II with standard criteria, including Bazett's formula for heart rate correction. Follow-up data included information on hospitalizations, arrhythmic events (e.g., ventricular tachycardia, fibrillation) and survival data, with follow-up visits scheduled every 3 months over a period of 1 year. The primary outcome was the change in QT interval over time and its association with changes in heart failure severity, measured by NYHA class, LVEF and BNP levels, while secondary outcomes included hospitalization rates, arrhythmic events and mortality. Data were analyzed using descriptive statistics to summarize baseline characteristics, Spearman's Rank Correlation to assess the relationship between QT interval prolongation and heart failure severity, linear regression to explore the relationship between QT interval changes and heart failure severity over time (adjusting for confounders) and Kaplan-Meier Survival Analysis to evaluate the impact of QT prolongation on hospitalization rates and mortality. For sample size calculation, the study assumed a moderate effect size (Cohen's d = 0.5) with 80% power and a significance level of 5%. The estimated sample size was 100-120 participants, with an additional 20% inflated for potential dropouts and non-compliance, resulting in a final sample size of 120-144 participants. Ethical approval was obtained from an institutional review board (IRB) and written informed consent was obtained from all participants, ensuring they understood the study's nature and their rights. Confidentiality and data protection measures were implemented to safeguard participant privacy.

## Results:

The results of the study were based on data collected from 130 heart failure patients who were followed for a period of 1 year. The patients were categorized into two groups: those with reduced ejection fraction (HFrEF) and those with preserved ejection fraction (HFpEF). Baseline characteristics, including demographic data, clinical measures of heart failure severity and ECG findings, are summarized in Table 1. At baseline, the study found that QT interval prolongation was significantly more pronounced in patients with HFrEF compared to those with HFpEF. The average QT interval in the HFrEF group was 455 ms, while in the HFpEF group; it was 420 ms (p < 0.05). Over the 1-year follow-up period, QT interval prolongation correlated significantly with worsening NYHA class, with an average increase of 10 ms in the QT interval for every 1-point increase in NYHA class (p < 0.01). This finding is shown in Table 2. The study found a significant negative correlation between QT interval prolongation and left ventricular ejection fraction (LVEF). As heart failure severity increased (evidenced by a lower LVEF), the QT interval prolonged. Specifically, for every 5% decrease in LVEF, the QT interval prolonged by 4.3 ms (r = -0.65, p < 0.001). This correlation is detailed in Table 3. There was a positive correlation between brain natriuretic peptide (BNP) levels and QT interval prolongation. As BNP levels increased, indicative of worsening heart failure, the QT interval also prolonged. The average QT interval in patients with BNP levels higher than 800 pg/mL was 470 ms, compared to 415 ms in those with BNP levels lower than 200 pg/mL (p < 0.05). This relationship is illustrated in Table 4. Patients with prolonged QT intervals had significantly higher rates of hospitalization and arrhythmic events. The incidence of ventricular tachycardia or fibrillation was 18% in patients with QT intervals greater than 460 ms, compared to 7% in those with shorter QT intervals (p < 0.05). Furthermore, patients with QT prolongation (>460 ms) had an average of 2.5 hospitalizations per year, compared to 1.4 hospitalizations for those with shorter QT intervals (p < 0.01). These results are presented in Table 5. After 1 year, the study observed that QT interval prolongation was associated with increased mortality. The 1-year mortality rate for patients with QT prolongation (QT > 460 ms) was 22%, compared to 10% in those with normal QT intervals (p < 0.05). This data is summarized in Table 6. A multivariate regression analysis was conducted to assess the independent association between QT prolongation and adverse outcomes, adjusting for age, gender, medication use, comorbidities and baseline heart failure severity. The analysis revealed that QT interval prolongation remained a significant independent predictor of worse outcomes, including mortality, hospitalization and arrhythmic events (p < 0.01). The results of this analysis are shown in Table 7.

## Discussion:

The findings of this study support the hypothesis that QT interval prolongation is associated with the severity of heart failure (HF), particularly in relation to clinical markers like NYHA class, LVEF and BNP levels. Our results also emphasize the prognostic value of QT prolongation, with associations between prolonged QT intervals and increased hospitalizations, arrhythmic events and mortality, which aligns with previous studies in this area. Elston *et al.* (2016) [13] found a significant relationship between QT interval prolongation and worse outcomes in heart failure patients. Their study showed that QT prolongation was associated with higher mortality and arrhythmic events in HF patients, particularly those with HFrEF. Our study corroborates these findings, highlighting QT prolongation as an important prognostic indicator for adverse outcomes in HF patients. Gotta and Donner (2025) [14] explored the role of QT prolongation in predicting arrhythmic events in heart failure, finding that QT interval was an independent risk factor for ventricular arrhythmias. Our study similarly found that prolonged QT intervals were associated with higher rates of arrhythmic events, especially in patients with LVEF <40%. Stoicescu *et al.* (2024) [15] reported that QT prolongation was correlated with lower LVEF and more frequent hospitalizations in heart failure patients. This study supports our findings that QT prolongation is linked to worsening heart failure severity and increased hospitalizations. Kallergis *et al.* (2012) [16] explored the pathophysiological mechanisms behind QT prolongation in heart failure, particularly the role of autonomic dysfunction and electrolyte imbalances. Our study's results also suggest that electrolyte disturbances may play a role in QT prolongation, especially in more severe cases of heart failure.

## Conclusion:

Our findings align with previous research that underscores the clinical significance of QT prolongation in heart failure. The correlation between QT interval prolongation and heart failure severity is evident and our results further suggest that QT prolongation serves as an independent predictor of adverse outcomes such as hospitalizations, arrhythmias and mortality. The consistency with prior studies reinforces the need for ongoing monitoring of the QT interval in heart failure patients as part of comprehensive management strategies.

## Figures and Tables

**Table 1:** Baseline characteristics of study population

**Characteristic**	**HFrEF (n=65)**	**HFpEF (n=65)**	**p-value**
Age (years)	64.2 ± 9.1	65.1 ± 8.4	0.58
Gender (M/F)	45/20	40/25	0.35
LVEF (%)	34.8 ± 8.3	55.2 ± 6.9	<0.001
NYHA Class III/IV (%)	48%	35%	0.12
QT Interval (ms)	455 ± 25	420 ± 20	<0.05

**Table 2:** Change in QT interval with worsening NYHA class

**NYHA Class**	**Baseline QT Interval (ms)**	**Follow-up QT Interval (ms)**	**p-value**
I	410 ± 15	415 ± 18	0.21
II	425 ± 18	435 ± 20	0.02
III	445 ± 20	460 ± 22	<0.01
IV	470 ± 25	485 ± 28	<0.01

**Table 3:** Correlation between QT interval and LVEF

**LVEF (%)**	**QT Interval (ms)**	**r-value**	**p-value**
<40	460 ± 30	-0.65	<0.001
40-50	440 ± 20	-0.42	<0.01
>50	410 ± 15	-0.15	0.12

**Table 4:** QT Interval and BNP Levels

**BNP (pg/mL)**	**QT Interval (ms)**	**p-value**
<200	415 ± 18	<0.05
200-800	440 ± 22	<0.05
>800	470 ± 25	<0.05

**Table 5:** Hospitalizations and Arrhythmic Events Based on QT Interval Prolongation

**QT Interval (ms)**	**Hospitalizations/Year**	**Ventricular Arrhythmias (%)**	**p-value**
<460	1.4	7%	<0.05
>460	2.5	18%	<0.01

**Table 6:** Mortality Rates Based on QT Interval

**QT Interval (ms)**	**1-Year Mortality Rate (%)**	**p-value**
<460	10%	<0.05
>460	22%	<0.05

**Table 7:** Multivariate analysis for predictors of adverse outcomes

**Variable**	**Hazard Ratio (HR)**	**95% CI**	**p-value**
QT Interval (ms)	1.05	1.02-1.08	<0.01
Age (years)	1.03	1.01-1.06	0.03
LVEF (%)	0.95	0.93-0.97	0.001
